# P-894. Impact of Extended Spectrum Beta Lactamase (ESBL) Culture Comment Removal on Antibiotic Prescribing Practices

**DOI:** 10.1093/ofid/ofaf695.1102

**Published:** 2026-01-11

**Authors:** Melissa Poulsen, Kazumi Morita, Daniel Mueller

**Affiliations:** Rutgers University, Philadelphia, PA; Temple University Hospital, Philadelphia, Pennsylvania; Temple University Hospital, Philadelphia, Pennsylvania

## Abstract

**Background:**

Since the Clinical Laboratory Standards Institute updated the susceptibility breakpoint for Enterobacterales in 2010, extended-spectrum beta-lactamase (ESBL) confirmatory testing is no longer required. Clinicians must instead interpret susceptibility patterns suggestive of ESBL production. This change may complicate antibiotic selection for bacteremia caused by ESBL-producing organisms, where carbapenems are preferred. This study evaluated the changes in antibiotic prescribing for ESBL organism bacteremia before (pre-cohort) and after (post-cohort) the removal of the ESBL identification comment.

**Methods:**

This retrospective, single center chart review included adult hospitalized patients who received ≥48 hours of definitive antibiotics for ESBL *Escherichia coli*, *Klebsiella pneumoniae*, or *Klebsiella oxytoca* bacteremia. The pre-cohort was identified by the ESBL comment in the culture report from 2/1/2017-12/31/2019. The post-cohort was identified by organisms with ceftriaxone resistance, a surrogate for ESBL production, in the culture report from 1/1/2021-10/31/2024. Polymicrobial infections and carbapenem-resistant organisms were excluded.

The primary endpoint was definitive carbapenem therapy within 48 hours from index culture susceptibility result. Key secondary endpoints included time to carbapenem order from index culture collection, *Clostridioides difficile* infection within 90 days, and 30-day all cause in-hospital mortality, recurrence, and readmission. Descriptive statistics, chi-squared, and t-test were used.

**Results:**

A total of 114 patients were included, 29 in the pre-cohort and 85 in the post-cohort. The percentage of patients who met the primary endpoint was 82.8% and 83.5%, respectively (p=0.923). In the post-cohort, five patients received definitive carbapenem therapy initiated at >48 hours compared to zero in the pre-cohort. The mean time to carbapenem order was 2.43 in the pre-cohort and 10.6 hours in the post-cohort (p< 0.001). There was no difference in clinical outcomes.

**Conclusion:**

Removal of the ESBL identification comment did not significantly impact the definitive use of carbapenems but did delay the time to carbapenem order. Antimicrobial stewardship interventions may improve the early recognition of ESBL-production.

**Disclosures:**

All Authors: No reported disclosures

